# A randomized controlled study on the efficacy and function of internal sphincter lowering combined with external sphincter denudation and virtual hanging drainage in the treatment of complex anal fistula

**DOI:** 10.3389/fsurg.2026.1726346

**Published:** 2026-04-10

**Authors:** Ling Wang, Xindan Zhang, Fuheng Liu, Ji Jin, Xiu Wang, Gang Zhao

**Affiliations:** 1 Department of Traditional Chinese Medicine, Suqian First People’s Hospital, Suqian, Jiangsu, China; 2 Department of Spleen and Stomach Diseases, Suqian Hospital of Traditional Chinese Medicine, Suqian, Jiangsu, China

**Keywords:** anal function, complex anal fistula, external sphincter denudation, internal opening downward displacement, randomized controlled trial, virtual hanging drainage

## Abstract

**Objective:**

The clinical treatment of complex anal fistula faces the dual challenges of cure rate and preservation of sphincter function. This study aims to evaluate the application effect of a novel surgical method - internal opening downward displacement combined with external sphincter denudation and virtual hanging drainage - in the treatment of complex anal fistula.

**Methods:**

A total of 102 patients with complex anal fistula confirmed by MRI were included in this study and randomly divided into the treatment group (internal opening downward displacement combined with external sphincter denudation and virtual hanging drainage) and the control group (traditional incision and hanging thread surgery). The surgical outcomes, changes in anal function (Wexner score), quality of life (EQ-5D score), and postoperative recurrence were compared between the two groups. The follow-up period was 12 months.

**Results:**

There were no significant differences in baseline data between the two groups. The treatment group had shorter operation time (69.22 ± 32.81 vs. 77.33 ± 40.66 min), shorter wound healing time (42.10 ± 3.65 vs. 47.54 ± 5.33 days), and shorter hospital stay (4.88 ± 1.84 vs. 9.94 ± 4.26 days) compared with the control group (all *P* < 0.001); the postoperative pain score was also significantly lower (2.88 ± 0.48 vs. 3.77 ± 0.83, *P* < 0.001). One month after surgery, the number of patients who recovered in the treatment group (40 cases) was more than that in the control group (37 cases, *P* < 0.05). The postoperative anal incontinence score in the treatment group was lower than that in the control group (1.35 ± 0.97 vs. 4.48 ± 1.23, *P* < 0.05). The complication rate in the treatment group was significantly lower than that in the control group (18.00% vs. 36.54%, *P* < 0.001). The recurrence rate at 1-year follow-up was only 2% in the treatment group and 7.69% in the control group (*P* < 0.05). The quality of life scores improved in both groups after surgery, but the improvement was more significant in the treatment group.

**Conclusions:**

The internal opening downward displacement combined with external sphincter denudation and virtual hanging drainage method shows good short-term and long-term efficacy in the treatment of complex anal fistula, balancing cure rate, fecal control function, and quality of life, and has a promising clinical application prospect.

## 1 Introduction

Anal fistula is a common and challenging condition in anorectal surgery, characterized by complex anatomy, a high recurrence rate, and substantial risk of postoperative anal sphincter dysfunction (1). Achieving effective fistula eradication while preserving continence remains a central dilemma in clinical practice. Traditional fistulotomy or incision with cutting seton drainage can achieve high healing rates, but these techniques are often associated with significant postoperative pain, prolonged wound healing, and an increased risk of anal incontinence due to progressive sphincter division (2, 3).

In recent decades, a variety of sphincter-preserving techniques have been introduced to address these limitations. Loose seton placement aims to maintain drainage while minimizing sphincter injury, but is frequently associated with prolonged treatment duration and variable recurrence rates (4). Procedures such as fistulotomy with sphincter reconstruction attempt to restore sphincter continuity but require advanced surgical expertise and may not be suitable for complex or high fistulas (5). The ligation of the intersphincteric fistula tract (LIFT) procedure offers an anatomically appealing solution by closing the internal opening without sphincter division; however, its effectiveness in complex fistulas remains inconsistent, with recurrence rates reported to be relatively high in some series (6, 7).

Despite these advances, no single technique has successfully resolved the inherent trade-off between fistula cure and sphincter preservation, particularly in patients with complex anal fistulas such as high trans-sphincteric or supra-sphincteric types (8). Moreover, many sphincter-preserving techniques are technically demanding and difficult to standardize, limiting their widespread adoption, especially in primary or secondary care settings.

Against this background, the Anorectal Department of Jiangsu Provincial Hospital of Chinese Medicine developed a modified surgical strategy termed internal opening downward displacement combined with external sphincter denudation and virtual hanging drainage. Rather than introducing a completely novel maneuver, this technique integrates three established principles—source control of the internal opening, maximal preservation of the external sphincter, and continuous but non-destructive drainage—into a single, standardized operative approach. The aim is to optimize the balance between eradication of infection, functional preservation, and wound healing.

The present randomized controlled study was designed to compare this combined technique with traditional incision and cutting seton drainage in patients with complex anal fistulas, with the objective of providing robust clinical evidence for its safety, efficacy, and potential value in routine surgical practice.

## 2 Materials and methods

### 2.1 Inclusion and exclusion criteria

This single-center randomized controlled study included 102 patients who visited the Anorectal Department of Suqian First People's Hospital between July 2022 and June 2023 and were diagnosed with complex anal fistulas based on pelvic MRI findings (9).

According to the widely accepted definitions in the literature, complex anal fistulas are characterized by high trans-sphincteric, supra-sphincteric, or extra-sphincteric fistula tracts, recurrent fistulas, multiple tracts, or fistulas associated with conditions such as inflammatory bowel disease or impaired sphincter function (10, 11). In particular, high trans-sphincteric fistulas are considered complex when a substantial portion of the external anal sphincter (EAS) is involved, due to the increased risk of postoperative fecal incontinence.

In the present study, fistula anatomy was evaluated using both MRI-based criteria and the Garg classification system. According to the Garg classification, fistulas were categorized into five grades based on the extent of sphincter involvement and fistula complexity. High trans-sphincteric fistulas and supra-sphincteric fistulas correspond to Garg grade III and grade IV fistulas (12, 13), which are considered complex fistulas requiring sphincter-preserving surgical strategies.

Complex anal fistulas eligible for inclusion were defined as high-level trans-sphincteric or supra-sphincteric fistulas, corresponding to Garg grade III or IV, as identified on pelvic MRI and confirmed intraoperatively.

High trans-sphincteric fistulas were defined as fistula tracts involving more than 30%–50% of the thickness of the external anal sphincter, in accordance with previous MRI-based studies and clinical guidelines (14, 15). The extent of EAS involvement was assessed on axial pelvic MRI images by measuring the length of the sphincter muscle traversed by the fistula tract and dividing it by the total thickness of the external anal sphincter at the same level.

The study protocol was reviewed and approved by the Ethics Committee of Suqian First People's Hospital (Approval No. 2025-SR-0334), and all procedures were performed in accordance with the Declaration of Helsinki (16). Written informed consent was obtained from all participants prior to enrollment.

The inclusion criteria were as follows: (1) age between 18 and 65 years; and (2) pelvic MRI indicating high-level trans-sphincteric or supra-sphincteric fistula tracts corresponding to Garg grade III or IV, as defined above. The exclusion criteria were as follows: (1) inflammatory bowel disease (including Crohn's disease and ulcerative colitis), due to its distinct pathophysiology and fistula behavior; (2) severe systemic diseases (such as severe cardiopulmonary, hepatic, or renal dysfunction) that could increase surgical risk or affect postoperative recovery; (3) pregnancy or lactation; (4) specific infections, including tuberculosis, HIV infection, or other systemic infectious diseases; (5) previous history of anal fistula surgery, as prior surgical intervention may result in scar formation and altered sphincter anatomy, potentially affecting surgical outcomes and functional evaluation; (6) malignant tumors of the anorectal region or other pelvic malignancies, which may independently influence healing and prognosis; (7) rectal prolapse, which can significantly affect anorectal anatomy and continence function; and (8) other significant anorectal diseases, such as severe hemorrhoids, anal stenosis, or anal fissures requiring surgical intervention, which could interfere with postoperative outcome assessment.

Eligible patients were randomly assigned to the treatment group (*n* = 50) or the control group (*n* = 52) using a random number table. The treatment group underwent internal opening downward displacement combined with external sphincter denudation and virtual thread-drawing drainage surgery, whereas the control group received traditional incision and thread-drawing surgery.

### 2.2 Randomization and allocation concealment

Eligible patients were randomly assigned to the treatment group or the control group in a 1:1 ratio. The random sequence was generated using a random number table by an independent researcher who was not involved in patient recruitment or outcome assessment (17).

The allocation sequence was concealed using sequentially numbered, sealed, opaque envelopes, which were opened only after patient enrollment and completion of baseline assessments. Due to the surgical nature of the interventions, blinding of surgeons and patients was not feasible; however, outcome assessments were performed according to predefined criteria to minimize bias.

Computer-based randomization software was not used in this single-center study because of the limited sample size and logistical constraints at the time of study initiation. More advanced randomization methods will be considered in future multicenter studies.

### 2.3 Preoperative assessment

All patients underwent a standardized preoperative assessment prior to randomization. This assessment included a detailed medical history and physical examination, with particular attention to anorectal symptoms, continence status, and previous anorectal conditions.

Pelvic MRI was performed in all patients to evaluate fistula anatomy, classify fistula type according to MRI-based criteria and the Garg classification, and assess the extent of external anal sphincter involvement.

Baseline continence function was assessed using the Wexner incontinence score, pain intensity was evaluated using the visual analog scale (VAS), and health-related quality of life was assessed using the EQ-5D questionnaire. All baseline assessments were completed before randomization to ensure comparability between the treatment and control groups.

### 2.4 Surgical methods

All procedures were performed by senior colorectal surgeons with extensive experience in the management of complex anal fistulas. Patients were placed in the lithotomy position under spinal or general anesthesia. Prophylactic antibiotics were administered perioperatively according to institutional protocols.

#### 2.4.1 Treatment group: internal opening downward displacement combined with external sphincter denudation and virtual hanging drainage

**Figure 1:**
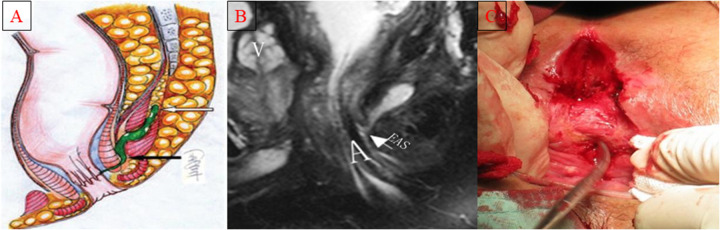
Schematic diagram of the operation for the internal opening of the deep posterior anal canal fistula to be lowered and the external sphincter to be exposed and hung for drainage. **(A)** Lateral view of the deep posterior anal canal fistula; **(B)** MR image of the deep posterior anal canal fistula; **(C)** Complete exposure of the external sphincter.

**Figure 2:**
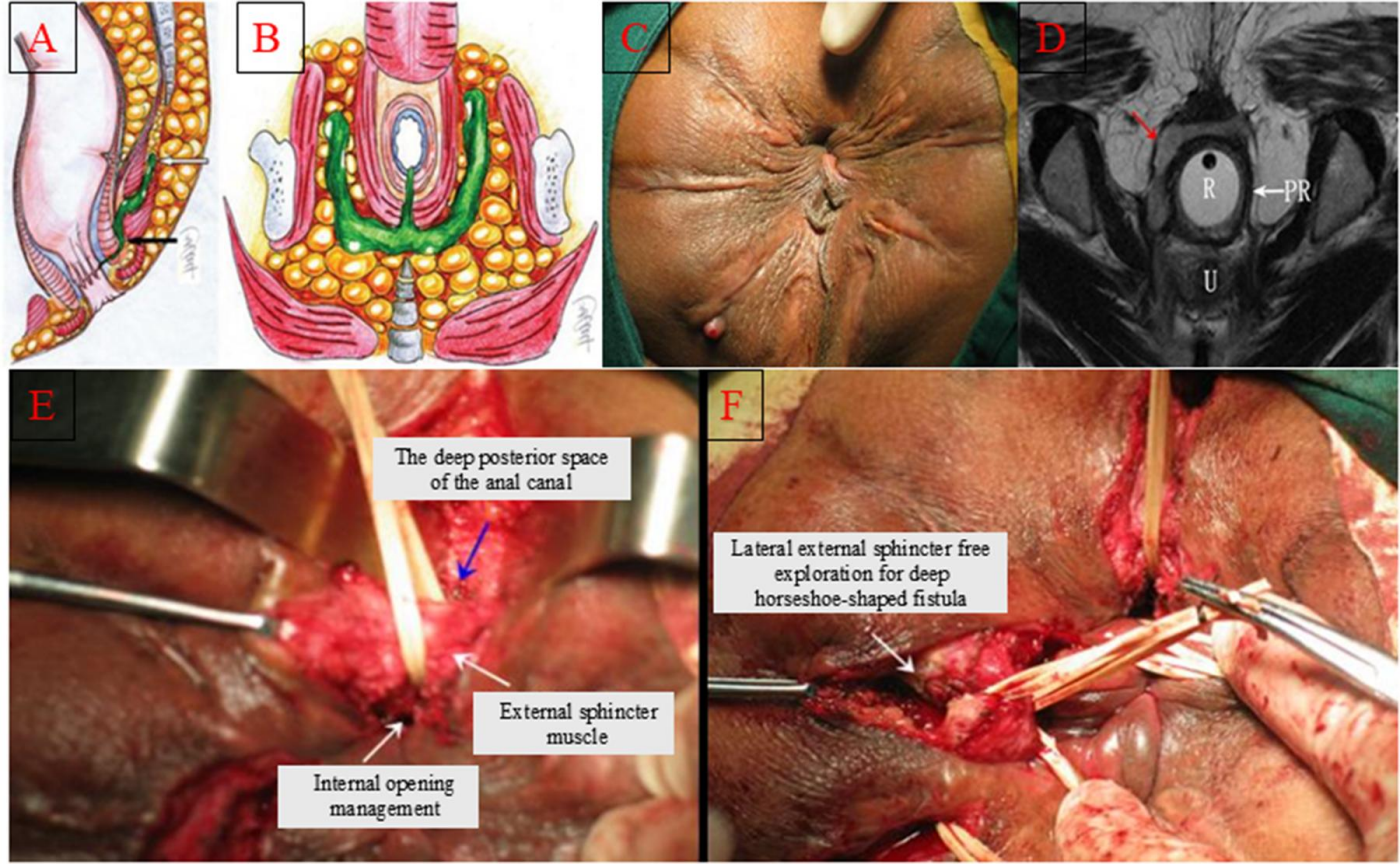
Schematic diagram of the operation for the internal opening of the horseshoe-shaped anal fistula in the deep posterior anal canal being shifted downward and the external sphincter being exposed and suspended for drainage. **(A)** Lateral view of the anatomy of the horseshoe-shaped anal fistula in the deep posterior anal canal; **(B)** Frontal view of the anatomy of the horseshoe-shaped anal fistula in the deep posterior anal canal; **(C)** Preoperative condition of the horseshoe-shaped anal fistula in the deep posterior anal canal; **(D)** MR of the horseshoe-shaped anal fistula in the deep posterior anal canal; **(E)** Complete exposure and suspension drainage of the external sphincter; **(F)** Suspended drainage between incisions of each branch.

(1) Identification of the fistula tract and internal opening: A malleable probe was gently introduced through the external opening to identify the direction of the fistula tract and confirm the location of the internal opening. The fistula anatomy identified intraoperatively was consistent with preoperative MRI findings. Internal opening downward displacement was selectively performed in patients with complex fistulas (e.g., high trans-sphincteric or supra-sphincteric fistulas) in whom the internal opening, although sometimes appearing at the dentate line, was functionally located within a high-pressure segment of the anal canal.
(2) Internal opening downward displacement: An incision was made along the probe from the external opening toward the internal opening, involving the skin and subcutaneous tissue. Fibrotic and inflamed tissue at the margin of the internal opening was excised. Subsequently, a limited portion of the internal anal sphincter and overlying mucosa was resected, extending approximately 0.5–1.0 cm distally from the original internal opening, while strictly remaining within the submucosal and superficial internal sphincter plane to avoid injury to the deeper sphincter complex and the anorectal ring. This maneuver resulted in relocation of the functional internal opening to a more distal position, typically 0.5–1.0 cm below the dentate line, at the level of the low-pressure anal canal or perianal skin. The newly created opening allowed improved drainage and reduced fecal contamination from the high-pressure anal canal.
(3) External sphincter denudation: From the external opening, the fistula tract was dissected along its course outside the external anal sphincter. The fistula wall adherent to the external sphincter was carefully separated, and the involved segment of the external sphincter was fully exposed and freed, while preserving the integrity of the sphincter muscle fibers. The remaining fistula wall was curetted to remove necrotic and inflammatory tissue, ensuring thorough debridement without transection of the external sphincter.
(4) Virtual hanging drainage: After adequate debridement, a sterile rubber band was placed beneath the freed external sphincter between the internal and external openings. Unlike a cutting seton, this rubber band was used solely for *virtual hanging drainage* and was not tightened postoperatively. Its purpose was to maintain continuous drainage and prevent premature closure of the superficial tract while preserving sphincter continuity.
(5) Drainage and wound management: The incision was extended as necessary to ensure unobstructed drainage. No intentional division of the external sphincter was performed. The wound was left open for secondary healing. The operative steps are illustrated in Figures 1, 2.

#### 2.4.2 Control group: traditional incision and cutting seton technique

(1) Identification of the fistula tract: Under probe guidance, the fistula tract was identified from the external opening to the internal opening. The tract was fully probed to confirm its course through the sphincter complex.
(2) Incision and seton placement: The skin and subcutaneous tissue overlying the fistula tract were incised. A cutting seton (rubber band) was passed through the fistula tract, encircling the involved portion of the sphincter complex.
(3) Seton tightening protocol: The seton was gradually tightened postoperatively at regular outpatient visits, typically every 7–10 days, depending on wound condition and patient tolerance. The tightening aimed to achieve slow transection of the sphincter muscle while allowing fibrosis and healing to occur simultaneously.
(4) Drainage and wound care: Additional incision or drainage was performed when necessary to ensure adequate discharge of purulent material. The seton was maintained until the fistula tract was completely transected and healed.

### 2.5 Observation indicators and evaluation criteria

(1) Intraoperative Indicators: Operation time (minutes);
(2) Short-term Postoperative Indicators: Wound healing time, hospital stay, VAS postoperative pain score, and incidence of complications (such as dysuria, incision infection, gas leakage, etc.);
(3) Functional Assessment: Wexner incontinence score (7) and EQ-5D quality of life score (8) before and after surgery;
(4) Efficacy Evaluation: According to the “Diagnostic and Therapeutic Standards for Traditional Chinese Medicine Diseases and Syndromes” (9), the results were classified as cured, markedly effective, effective, and ineffective;
(5) Long-term Indicators: Recurrence rate and functional score changes at 6 and 12 months postoperatively.

### 2.6 Follow-up schedule

All patients were followed up according to a predefined postoperative protocol. Follow-up visits were scheduled at 1 week, 1 month, 3 months, 6 months, and 12 months after surgery. The assessments performed at each time point are summarized below:

**Postoperative 1 week:** (1) Wound inspection and drainage assessment; (2) Evaluation of postoperative pain using VAS; (3) Recording of early complications (e.g., bleeding, infection).
**Postoperative 1 month:** (1) Assessment of wound healing status; (2) Evaluation of pain (VAS); (3) Recording of postoperative complications.
**Postoperative 3 months:** (1) Assessment of wound healing; (2) Continence evaluation using the Wexner incontinence score; (3) Quality-of-life assessment using the EQ-5D questionnaire.
**Postoperative 6 months:** (1) Continence evaluation (Wexner score); (2) Quality-of-life assessment (EQ-5D) (3) Clinical evaluation for fistula recurrence.
**Postoperative 12 months:** (1) Final assessment of recurrence; (2) Continence function (Wexner score); (3) Quality-of-life assessment (EQ-5D); (4) Pelvic MRI performed when recurrence was clinically suspected.

All follow-up data were collected prospectively and recorded using standardized case report forms.

### 2.7 Sample size calculation

The sample size was calculated based on the primary outcome of wound healing time, which was considered clinically relevant according to previous studies and preliminary clinical observations.

Assuming a two-sided significance level (α) of 0.05 and a statistical power (1−β) of 80%, a clinically meaningful difference in wound healing time between the two groups was used for estimation, together with the expected standard deviation. The sample size calculation was performed using standard formulas for comparison of two independent means and confirmed using statistical software.

The estimated minimum sample size required was at least N patients per group. Considering potential loss to follow-up, a total of 102 patients were ultimately enrolled in the study (50 in the treatment group and 52 in the control group), which met the sample size requirement.

### 2.8 Statistical methods

All data were analyzed using SPSS 19.0 statistical software. Count data were analyzed using the χ^2^ test; measurement data were first subjected to normality and homogeneity of variance tests. If they conformed to a normal distribution, they were expressed as (x¯ ± S), and paired sample *T*-tests were used for comparisons within groups, while independent sample *T*-tests were used for comparisons between groups. If they did not conform to a normal distribution, they were expressed as [M(Q25, Q75)] and non-parametric tests were used.

## 3 Results

### 3.1 Comparison of general data between the treatment group and the control group

Table 1 shows that there were no statistically significant differences between the treatment group and the control group in terms of gender, age, disease duration, smoking status, and past medical history (*P* > 0.05).

**Table 1:** Comparison of general data between the treatment group and the control group [*n* (%) or $\bar{x}\;\;\pm \;s$].

Group	Age (years)	Gender (Male)	Disease duration (months)	Smoking (cases)	Previous surgery (yes)
Control group (*n* = 52)	37.67 ± 10.07	44 (84.62)	3.38 ± 2.49	16 (30.77)	15 (28.85)
Treatment group (*n* = 50)	3,612 ± 9.52	39 (78.00)	3.41 ± 2.54	18 (36.00)	17 (34.00)
*t/x^2^* value	0.607	0.738	0.126	0.089	0.234
*P*-value	0.437	0.121	0.773	0.928	0.539

### 3.2 Comparison of clinical indicators between the treatment group and the control group

Both groups of patients successfully completed the surgery. The operation time, wound healing time, and hospital stay of the treatment group were shorter than those of the control group, and the postoperative pain degree was lower than that of the control group. All differences were statistically significant (*P* < 0.05). See Table 2.

**Table 2:** Comparison of clinical indicators between the treatment group and the control group [*n* (%) or $\bar{x}\;\;\pm \;s$].

Group	Operation time (minutes)	Wound healing time (days)	Hospital stay (days)	Postoperative pain degree (points)
Control group (*n* = 52)	77.33 ± 40.66	47.54 ± 5.33	9.94 ± 4.26	3.77 ± 0.83
Treatment group (*n* = 50)	69.22 ± 32.81	42.10 ± 3.65	4.88 ± 1.84	2.88 ± 0.48
*t/x^2^* value	4.387	2.738	7.385	3.839
*P*-value	<0.001	<0.001	<0.001	<0.001

### 3.3 Comparison of anal function and anorectal dynamics before and after surgery between the treatment group and the control group

There was no statistically significant difference in the Wexner score between the treatment group and the control group before surgery [0.73 ± 0.68 vs. 0.75 ± 0.64] (*P* > 0.05). After surgery, the Wexner score of both groups increased (*P* < 0.05), and the Wexner score of the treatment group (1.35 ± 0.97) was significantly lower than that of the control group (4.48 ± 1.23), with a statistically significant difference (*P* < 0.05).

### 3.4 Comparison of clinical efficacy and complications between the treatment group and the control group

One month after treatment, the number of patients who were cured in the treatment group was significantly higher than that in the control group (*P* < 0.05), while there was no statistically significant difference in the total effective rate between the two groups (*P* > 0.05). The number of patients with dysuria after surgery in the control group was significantly higher than that in the treatment group (*P* < 0.05), and the total incidence of complications in the control group was higher than that in the treatment group, with a statistically significant difference (*P* < 0.05). For details, please refer to Table 3.

**Table 3:** Comparison of clinical efficacy and complications between the treatment group and the control group [*n*(%) or $\bar{x}\;\;\pm \;s$].

Group	Complications				Overall incidence rate (%)	therapeutic effect				Overall efficiency (%)
	Urination difficulty	Air and fluid leakage	Anal deformation	Incision infection		Cured	Markedly effective	Effective	Invalid	
Control group (*n* = 52)	13	2	1	3	19 (36.54)	37	5	6	4	48 (92.31)
Treatment group (*n* = 50)	5	1	1	2	9 (18.00)	40	7	2	1	49 (98.00)
*t/x^2^* value					7.385					1.698
*P*-value					<0.001					0.008

### 3.5 Comparison of quality of life before and after surgery between the treatment group and the control group

Before surgery and at 12 months after surgery, there was no significant difference in the scores of physical function, mental health, social function, and cognitive function between the two groups (*P* > 0.05); at 6 months after surgery, the scores of physical function, mental health, social function, and cognitive function in the treatment group were all higher than those in the control group (*P* < 0.05). See Table 4.

**Table 4:** Comparison of quality of life before and after surgery between the treatment group and the control group [ $\bar{x}\;\;\pm \;s$].

Group	Mental health			Cognitive function			Physiological function			Social functions		
	Preoperative	1 month postoperative	12 months postoperative	Preoperative	1 month postoperative	12 months postoperative	Preoperative	1 month postoperative	12 months postoperative	Preoperative	1 month postoperative	12 months postoperative
Control group (*n* = 52)	64.0 ± 2.3	68.0 ± 1.6*	70.88 ± 1.51*	64.0 ± 2.8	67.9 ± 1.5*	70.98 ± 1.30*	63.3 ± 2.4	67.9 ± 1.5*	70.82 ± 1.82*	63.6 ± 2.5	67.9 ± 1.4*	70.14 ± 1.52*
Treatment group (*n* = 50)	63.5 ± 2.38	70.51 ± 1.41*	71.76 ± 1.38*	63.6 ± 2.29	70.20 ± 1.39*	71.55 ± 1.35*	63.72 ± 2.29	70.02 ± 1.64*	71.12 ± 1.05*	63.26 ± 2.1	70.35 ± 1.60*	71.31 ± 0.93*
*t/x^2^* value	0.236	2.137	1.298	0.251	2.381	0.893	0.044	3.049	0.983	0.071	3.287	1.087
*P*-value	0.816	0.003	0.094	0.803	0.001	0.127	0.966	<0.001	0.103	0.958	<0.001	0.102

* Indicates that the difference is statistically significant.

### 3.6 Postoperative recurrence in the treatment group and the control group

One-year follow-up after discharge was conducted to record the recurrence of patients in the treatment group and the control group. The results showed that there was 1 recurrence in the treatment group, with a recurrence-free rate of 98%, while there were 4 recurrences in the control group, with a recurrence-free rate of 92.31%. The difference in recurrence-free rates between the two groups was statistically significant (x^2^ value = 11.438, *P* < 0.05).

## 4 Discussion

The main findings of this study indicate that internal opening downward displacement combined with external sphincter denudation and virtual hanging drainage offers several advantages in the treatment of complex anal fistula. Compared with traditional incision and cutting seton drainage, this approach significantly shortened operative time, promoted faster wound healing, reduced hospital stay and postoperative pain, lowered the incidence of postoperative anal incontinence and complications, and achieved a lower recurrence rate within one year. These results suggest that the proposed technique provides a more favorable balance between therapeutic efficacy and functional preservation (4).

Several surgical techniques reported in the literature share partial similarities with components of our approach, yet differ substantially in principle and execution. Cutting seton techniques prioritize fistula eradication through gradual sphincter division, often at the cost of continence impairment (18, 19). Loose seton placement reduces sphincter injury but may fail to adequately control the internal opening, leading to prolonged treatment courses and recurrence (4, 20). Recent evidence by Tomasicchio et al. also emphasized that even in low trans-sphincteric fistulas, seton-based management can be associated with prolonged treatment duration and inconsistent healing, further highlighting the limitations of seton therapy in complex scenarios (21). Fistulotomy with sphincter reconstruction attempts to restore anatomy but requires advanced expertise and carries a risk of dehiscence or functional deterioration (22, 23). The LIFT procedure focuses on closure of the internal opening within the intersphincteric plane, but its effectiveness in complex fistulas remains variable, particularly when secondary tracts or extensive fibrosis are present (24, 25).

The innovation of the present technique does not lie in the invention of a single new maneuver, but rather in the intentional and synergistic combination of three complementary strategies. First, internal opening downward displacement addresses the primary infectious source by relocating it from a high-pressure anal canal environment to a lower-pressure region. Second, external sphincter denudation allows thorough tract clearance while preserving the integrity of the sphincter muscle fibers. Third, virtual hanging drainage ensures continuous drainage without the progressive sphincter injury inherent to cutting setons. Together, these elements create a “1 + 1 + 1 > 3” effect, achieving adequate source control, functional preservation, and optimal drainage simultaneously (4).

Although the internal opening in some cases appeared to be located at the dentate line, the rationale for internal opening downward displacement lies in altering the pressure dynamics of the fistula rather than merely changing its anatomical level. The dentate line does not represent a strict functional boundary of anal canal pressure. In complex fistulas, particularly high trans-sphincteric or supra-sphincteric types, the internal opening often remains within a relatively high-pressure environment influenced by internal sphincter tone (26, 27). By relocating the functional internal opening to a more distal, lower-pressure region, fecal contamination is reduced, drainage is improved, and the healing environment of the fistula tract is optimized, thereby reducing the risk of recurrence.

The longer operative time and delayed wound healing observed in the control group can be explained by the need for extensive probing, multi-point seton placement, and the continuous mechanical irritation caused by cutting setons. These factors have also been reported in previous studies and are consistent with the known disadvantages of traditional seton techniques (18, 19).

From a clinical perspective, the greatest value of this technique lies in its ability to resolve a long-standing contradiction in fistula surgery: achieving reliable cure without sacrificing sphincter function. This makes it particularly suitable for patients with high functional preservation demands, such as women, elderly individuals, and patients with borderline continence.

Several limitations should be acknowledged. This was a single-center study, and surgeon-specific operative habits may have influenced outcomes. In addition, although follow-up was systematic, the duration was limited to one year. Future multicenter studies with standardized operative protocols, video documentation, and longer follow-up are needed to confirm the generalizability of these findings.

In conclusion, internal opening downward displacement combined with external sphincter denudation and virtual hanging drainage represents a rational and clinically valuable evolution of existing surgical concepts rather than a simple variation of known techniques. By integrating source control, sphincter preservation, and non-destructive drainage into a unified approach, this technique provides a promising option for the management of complex anal fistulas.

## Data Availability

The original contributions presented in the study are included in the article/Supplementary Material, further inquiries can be directed to the corresponding author.
